# Insertional Mutagenesis Approaches and Their Use in Rice for Functional Genomics

**DOI:** 10.3390/plants8090310

**Published:** 2019-08-29

**Authors:** Hasthi Ram, Praveen Soni, Prafull Salvi, Nishu Gandass, Ankita Sharma, Amandeep Kaur, Tilak Raj Sharma

**Affiliations:** 1 Department of Agri-Biotechnology, National Agri-Food Biotechnology Institute (NABI), Sector-81, SAS Nagar, Mohali 140306, India; 2 Department of Botany, Rajasthan University, Jaipur 302004, India

**Keywords:** insertional mutagenesis, rice, T-DNA, transposons, functional genomics

## Abstract

Insertional mutagenesis is an indispensable tool for engendering a mutant population using exogenous DNA as the mutagen. The advancement in the next-generation sequencing platform has allowed for faster screening and analysis of generated mutated populations. Rice is a major staple crop for more than half of the world’s population; however, the functions of most of the genes in its genome are yet to be analyzed. Various mutant populations represent extremely valuable resources in order to achieve this goal. Here, we have reviewed different insertional mutagenesis approaches that have been used in rice, and have discussed their principles, strengths, and limitations. Comparisons between transfer DNA (T-DNA), transposons, and entrapment tagging approaches have highlighted their utilization in functional genomics studies in rice. We have also summarised different forward and reverse genetics approaches used for screening of insertional mutant populations. Furthermore, we have compiled information from several efforts made using insertional mutagenesis approaches in rice. The information presented here would serve as a database for rice insertional mutagenesis populations. We have also included various examples which illustrate how these populations have been useful for rice functional genomics studies. The information provided here will be very helpful for future functional genomics studies in rice aimed at its genetic improvement.

## 1. Introduction

Inheritable changes in the DNA sequence that arise due to a replication error or as a result of a mutagen are known as mutations, which serve as a key force of variations and evolution of an organism. Researchers have used various mutagenesis approaches to increase variations in populations in order to study the evolutionary process [1]. Additionally, induced mutagenesis has also been used for the genetic improvement of many organisms, including microbes, animals, and plants. In agriculture, mutations have been successfully used for developing many new varieties with improved agronomic characteristics, such as higher stress tolerance potential (biotic and abiotic stress), bio-fortification, etc. [2]. Mutation approaches are also being used to study the function of genes in plant biology through both forward and reverse genetics approaches [3]. Various other gene targeting strategies using loss/gain of function of candidate gene(s) have been widely used to analyze gene functions. These approaches are collectively called functional genomics approaches.

The available approaches for inducing mutations include the use of chemicals or physical or biological agents [4]. The chemical mutagens include N-methyl-N-nitrosourea (MNU), sodium azide, hydrogen fluoride (HF), methyl methanesulfonate (MMS), or ethyl methanesulfonate (EMS), while the physical mutagens comprise fast neutron, UV, and X-ray radiations. Agrobacterium and transposon-based chromosomal integration are considered as an important biological mutagen [5]. Recent advents in genome editing approaches have revolutionized functional genomics studies by providing a straightforward way to precisely engineer a desired mutation in the genome. Among approaches like TALEN (transcription activator-like effector nuclease) or ZNF (zinc finger nuclease), the CRISPR (clustered regularly interspaced short palindrome repeats) is one of the most promising tools for genome engineering due to its high efficiency along with its specificity and simplicity [6].

EMS (ethyl methanesulfonate), a mono-functional ethylating agent, is a highly effective and commonly used method for generating mutant populations [7]. The EMS population provides the principal advantage of being an important functional genomics tool; it also has practical uses in crop breeding due to its ability to induce dominant mutant alleles and the ease with which it can be used. EMS alkylates guanine bases and forms O6-ethylguanine that pairs with thymine (T) instead of cytosine (C), resulting in C/G to T/A transitions. However, EMS may also cause G-C to C-G or G-C to T-A transversions by 7-methylguanine hydrolysis, albeit at lower frequency [8]. Therefore, by generating loss or gain of function mutants, EMS can be used to assess the functional role of single amino acid residues. EMS mutagenesis appears to be equally effective against crops with varying genome sizes, as similar mutation rates were observed in *Arabidopsis* and maize despite having a difference of approximately 20-fold in the genome size [9]. Thus, the EMS mutagen is preferred over the TILLING (targeting induced local lesion in the genome) approach in plants. It is a reverse genetic approach combined with chemical mutagenesis that facilitates the screening of point mutation by a mismatched cleavage in heteroduplex DNA with the endonuclease (CEL-I) [10]. After its first application for *Arabidopsis thaliana* mutant collection, the EMS-induced TILLING population has been used to develop a large number of organisms, including *Caenorhabditis elegans*, *Drosophila*, rice, wheat, barley, maize, *Brassica*, *Medicago truncatula*, etc. The interaction of electromagnetic (EM) spectrum of radiation with the atom dislodges electrons from the nuclear orbits and ionizes the impacted atoms. Ionizing radiations, such as fast neutrons, are used to cause larger deletions in the genome, from a few base pairs to several kilobases. Fast neutron bombardment has been effectively used to disrupt small genes or tandemly-arrayed genes to generate rice mutants. However, this process needs expensive instruments, a specialized laboratory setup, and a large sample size. Furthermore, these mutations are genetically less stable [11]. Despite a high mutation rate being caused by physical mutagens, it usually results in large chromosomal segment aberrations or the deletion of linked genes, which makes it less appropriate for gene mapping.

Among all the induced mutagens, mutagenesis by T-DNA and transposons are most widely used for large-scale analyzes in rice. In recent years there has been remarkable progress in the generation of insertional mutant populations [12,13]. Here we review recent progress in the application of insertion mutagenesis for functional genomics studies and compile available T-DNA mutant resources in rice. This collection of a large number of insertional mutant populations will accelerate functional genomics studies in this model monocot species.

## 2. Insertional Mutagenesis

Insertional mutagenesis is an important genetic tool for gene discovery using T-DNA, retrotransposons, activator/dissociation (Ac/Ds) insertions, and transposons. Insertional mutagenesis has been broadly employed to create mutant libraries that allow for the easy identification of tagged genes by use of PCR-based techniques, such as thermal asymmetric interlaced PCR(TAIL-PCR) or Inverse PCR. Additionally, the identification of biological functions for redundant or vital genes is not feasible with the use of knockout mutants generated using chemical or physical mutagens. Besides, insertional mutagenesis is a key tool to overcome such concerns, along with the use of activation tagging or the gene trap approach [14]. The rapidly-evolved cost-efficient high-throughput Next Generation Sequencing (NGS) platforms filled the gap of comprehensive mutant collections and characterized mutant collections for the systematic evaluation of gene function. In the insertional mutagenesis approach, the strict requirement of plant transformation methods and the relatively low frequency of mutations are the major limiting factors for the generation of large mutant populations. However, improvements in the efficient rice transformation protocol and advance vector repertoire availability have made it an important tool for genetic studies in a particular genetic background. In rice, the insertional mutagenesis approach is majorly attained in japonica cultivar due to the availability of reliable and reproducible *Agrobacterium tumefaciens*-mediated transformation (ATMT) methods [15]. Here the mutagen acts as a molecular tag and facilitates the identification of disrupted genes. The association of the insertion tag along with the desirable phenotype helps to isolate the gene of interest. The molecular tags used for insertional mutagenesis can be of different types, such as T-DNA, Activator element/Dissociation element (Ac/Ds), transposon, or retrotransposon.

## 3. T-DNA Insertional Mutagenesis

**Figure 1:**
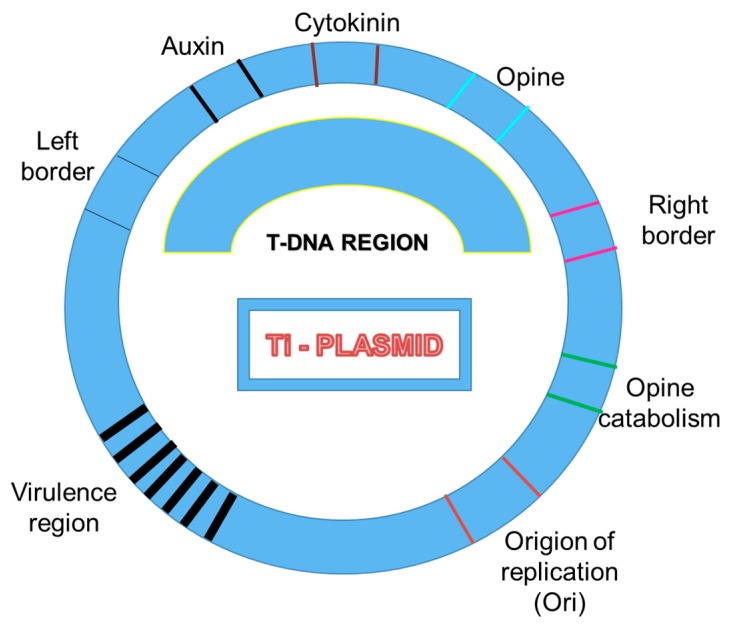
Major characteristics of a Ti-plasmid. Different important DNA regions are highlighted here.

*Agrobacterium tumefaciens*, a causative organism of crown gall disease, is a gram-negative bacterium which transfers a part of DNA, which is termed as T-DNA, from its tumor-inducing (Ti) plasmid into the host genome. T-DNA fragment is enclosed by two 25 base-pairs (bp) imperfect direct repeat borders which are eventually transferred into the plant genome, whereas another region present in Ti-Plasmid, known as the *vir* region, is comprised of seven operons (*virA*, *virB*, *virC*, *virD*, *virE*, *virF,* and *virG*) which encode proteins required to support proper processing of T-DNA insertion (Figure 1). In the T-DNA insertional mutagenesis approach, the T-DNA is delivered into the plant cells by co-culturing the cells with *Agrobacterium tumefaciens* culture. Functional analysis of the rice genome at a large scale is accelerated by random insertional mutagenesis by T-DNA. T-DNA insertions offer a high frequency and stable phenotype with rapid genetic screening of tagged genes. The identification of tagged lines is based on either PCR or by searching the Flanking Sequence Tag (FST) database. Recently, researchers have documented the use of T-DNA mutants to decipher the role and regulation of several genes. For instance, Ning et al. characterized a rice T-DNA insertion mutant *ILA1* (increased leaf angle1), which is a regulator for formation of mechanical tissue at the leaf joint. The abnormalities of T-DNA insertion mutant in the mechanical tissue formation leads to increased leaf inclination angle and altered cell wall composition in the leaf lamina joint. Two important genes involved in floral development, *RID1* (master switch for floral induction) and *JMJ706* (regulate flower development), were also identified through the T-DNA mutagenesis approach [16,17]. *JMJ706* encodes a demethylase (H3K9) that regulates the expression of genes involved in floral morphology and organ numbers in rice. *Rice Indeterminate 1* regulates the expression of a subset of regulatory genes including *Ehd1* (*Early heading date 1*), *Hd3a* (a rice ortholog of *FT*), and *RFT1* (*Rice flowering locus T1*) for controlling flowering. T-DNA insertion in the gene encoding β-carotene hydroxylase (BCH) harbours a phenotype hypersensitive to drought and oxidative stress. The *DSM2* gene precisely controls the ABA synthesis and xanthophyll cycle, which plays a decisive role in drought stress endurance in rice. Moreover, T-DNA mutagenesis exhibits low-copy number integration (average of two copies at 1.4 loci per line), which demands a large library construction to ensure appropriate genome coverage [18].

## 4. Transposon Integration Mutagenesis

Transposons, also known as jumping genes, are small mobile DNA sequences that are capable of moving to different positions within the genome. Transposons create transient break in double standard (ds) DNA followed by successive fusion of dsDNA. These small DNA sequences (500–1500 bp long) are also referred to as molecular parasites due to their ability to ensure their own replication with the help of several host factors [19]. However, sometimes transposons may impart beneficiary functions to the host organism and may turn its parasitism into symbiosis. One significant example is the spread of antibiotic resistance genes via transposable elements [20]. Approximately 35% of the rice genome is composed of transposon-derived sequences (International Rice Genome Sequencing Project, 2005). Active transposons are regarded as a menace to the host genome as they trigger the events like chromosomal rearrangements and genetic changes. A few genetic elements which flee from the genetic or epigenetic control of the cell are considered as active transposons and may endow a plant with a mutant phenotype. Although T-DNA mutagenesis is preferred over the transposon approach in rice due to the amenability of transformation protocol, however, in some cases, the transposon approach offers a reasonable advantage over the former. For instance, the transposon usually generates a single copy insertion compared to T-DNA, which tends to be integrated into multiple arrays. Furthermore, unlike T-DNA insertion, it is relatively easy to remobilize the tag from transposon tagged mutant lines to remove the transposon tag and produce a wild-type revertant. Finally, requirement of a comparatively smaller parental line to generate a large number of transposon tagged mutant population endows an advantage to its acceptability over T-DNA tagging, despite the immense labor involved [21]. 

Transposon elements were first identified in the late 1940s by Barbara McClintock in maize, but are now known to be present in almost all organisms. Transposons are categorized into two classes on the basis of their transposition mechanisms [22]. Class I transposable elements (TEs) or retrotransposons mobilize in a “*copy-and-paste”* manner through RNA intermediate, while Class II or DNA transposons harness the “*cut-and-paste*” mode of transposition. In both Class I and Class II TEs, transposition can further be distinguished into autonomous or non-autonomous based on the requirement for another TE to move. The movement of transposon can be controlled by splitting the transposase source from the transposable DNA, which will result in a non-autonomous TE. For example, the activator element (*Ac*) is an autonomous TE, and a dissociation element (*Ds*) is a non-autonomous TE. The transposition of *Ds* is essentially dependent on the presence of *Ac*. The two-component transposon systems from maize, *Ac/Ds* and *En/Spm-dSpm* (Enhancer/Suppressor Mutator), have been used to develop large scale mutant populations in rice [23]. This approach has attained notable success in rice to tag and isolate genes involved in several key cellular processes. For instance, a semi-dwarf mutant in rice has been identified by the phenotypic screening of *Ds* insertion lines. The integration of *Ds element* into *OsCYP96B4* (*Oryza sativa Cytochrome P450 96B4*) gene resulted in defective in cell elongation and pollen germination with semi-dwarf phenotype [24]. Similarly, insertion of the *Ds element* into the *Osnop* (*Oryza sativa no pollen*) gene resulted in a sterile male mutant plant with pollen-less flowers. On the other hand, rice-based transposons such as *miniature Ping* (*mPing*) and *non-autonomous DNA-based active rice transposon* (*nDart1*) have been successfully adopted for the functional analysis of rice genes. *mPing* is a 430 bp active miniature inverted-repeat transposable element (MITE) which was initially identified in rice [25,26,27].

The MITEs usually exhibit high copy numbers in the genome, however, the copy number of *mPing* elements is remarkably low in most rice cultivars. *mPing* is a non-autonomous deletion derivative of the *Ping element* which lacks the coding capacity for transposition [28,29]. *nDart1* is a non-autonomous element which harbors active transposition with the coexistence of an autonomous element, *aDart1*. The *aDart1*-based tagged lines have been effectively used to develop mutant collections in rice [30]. On the other hand, an endogenous retrotransposon *Tos17* has been deployed to generate knockouts mutants in rice through reverse genetics approach [31].

## 5. Entrapment Tagging

Entrapment tagging is a powerful tool to discover novel genes and regulatory modules in rice. The trapped line can be used to assess the biological role of a gene as well as of the regulatory modules like enhancer or promoter. The insertional mutagens (T-DNA or TEs), combined with a gene or enhancer trap, help to improve the detection of the tagged gene. The trapped tagged lines have been successfully used to generate mutant libraries in rice. Entrapment tagging can be comprised of gene trap, promoter trap, and enhancer trap. In the enhancer traps (ET) strategy, a reporter gene (GUS/GFP) attached to a basal promoter (TATA box and a transcriptional start site) is integrated randomly into the genome. The reporter gene is activated only when the integration occurs in the vicinity of an enhancer element. The reporter gene expression is independent of the orientation of the disrupted gene. In promoter traps and gene traps, a promoter-less reporter gene is integrated in frame with a transcriptional unit which allows the expression of the reporter gene. Therefore, the vector needs to be inserted in the same orientation as the trapped gene. For generating transcriptional fusion, the integration site is usually needed to be at exon and intron for promoter trap and gene trap, respectively. In addition, in the gene trap (GT) assembly, preceding the reporter gene there is an intron with multiple splice acceptor sites, which share the splicing donor sites from the downstream chromosomal gene, resulting in a fusion of upstream exon to the reporter gene. The integrated reporter gene offers an easy and direct means to analyze the regulatory component that control gene expression [14]. Ac/Ds based gene trapping system has been targeted to the rice genome by *Agrobacterium*-mediated transformation and has been studied using the GUS reporter gene [32]. Similarly, for the development of enhancer trapped tagged lines in japonica rice, *GAL4/VP16-UAS* elements with GUS have been effectively used [18].Lee et al., 2004 [21], have used GUS-trapped T-DNA tagging lines for identification of stress-responsive genes in rice. A chlorophyll-deficient mutant (*OschlH*) with chlorina phenotype has also been identified using promoter-less GUS reporter gene in rice [21,33]. 

## 6. Screening of Insertional Mutants

**Figure 2:**
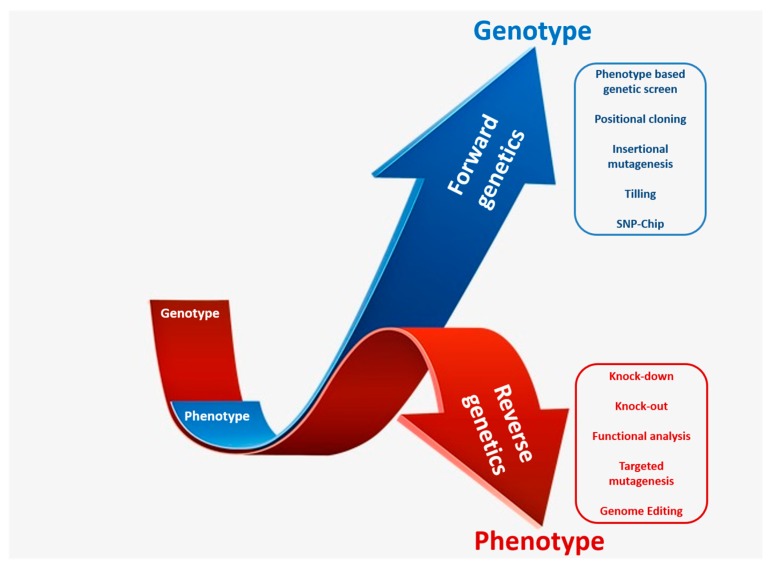
A schematic representation of forward and reverse genetic approaches for identification and characterization of candidate genes. Forward genetics studies start with the selection of the desired phenotype and culminate with the identification of the responsible gene responsible (depicted in blue arrow). Reverse genetics studies start with the selection of gene of interest and end with the analysis of resulting phenotype (depicted in red arrow).

The generation of mutant population demands immense labor, resource, and time; therefore, an appropriate screening method is essential for genetic analysis. Forward and reverse genetic screening are two important approaches for genetic analysis of genes and complex regulatory pathways associated with desired phenotypes (Figure 2). Large-scale screening of mutant collections has been deployed for the screening of many agronomically important traits.

Forward genetics approach is usually used for determining the genetic basis of a particular phenotype. Usually, a selectable marker is inserted into the genome, which is used to identify the mutagenized individuals. Furthermore, since a fragment of DNA with known sequence is inserted, it helps in mapping and cloning of the gene. Gene knockout mutations, which are usually recessive, show phenotype changes only in homozygous lines, whereas gene activation mutations are usually dominant in nature, and their phenotype changes are usually present in both homozygous and heterozygous lines. Mutant phenotypes caused by either gene knockout or gene activation are identified by comparing with wild-type individuals. Many rice genes, like *OsMADS50* and *OsDMKT1*, were isolated from tagged T-DNA lines using forward genetics [33]. To improve the applicability of forward genetics in rice, it will be important to develop new transformation techniques without tissue culture procedures.

Reverse genetics refers to the processes opposite to the direction of so-called forward genetics screens. It is used to study gene function, and this is a direct approach to eliminate and alter gene functions. In these approaches, mutations are first generated in a target gene, and then their phenotypes are investigated later on. Furthermore, multiple mutants are also generated by genetic crossing for further assessment. Reverse genetic approaches have also been performed with homologous recombination [34], antisense or RNAi suppression, and insertional mutagenesis [35,36]. Among various methods, random insertional mutagenesis by T-DNA and transposons are routinely used for large-scale analyzes. This technique is mainly used for identifying knockout mutants, but can also be employed for promoter trapping and activation tagging. The recent establishment of a large number of insertional mutant populations will accelerate functional genomics studies in rice (Table 1).

The process of reverse genetics has been utilized to screen T-DNA insertions in an increasing number of genes involved in signal transduction [45] and ion transport. Use of populations with transposons for reverse genetics has also been recently reported (Table 1). For example, insertion mutant lines with the *Enhancer/Suppressor-mutator* (*En/Spm*) transposon insertions in genes involved in flavonoid biosynthesis and gravitropism have been reported. Reverse genetics is particularly useful for functional genomics studies aimed at individual members of gene families.

## 7. Rice T-DNA Mutant Databases and Tools

**Figure 3:**
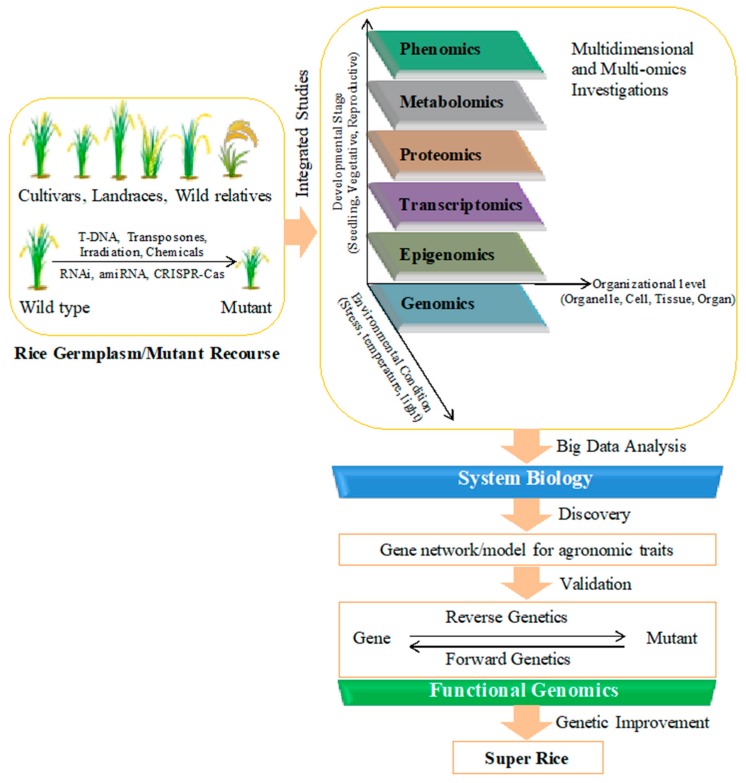
Systematic research approach involving integrated studies to develop super rice. Mutants lines generated through different mutagenesis techniques enrich the available rice resources along with the naturally occurring diversity of rice germplasm. Comprehensive analysis of available recourses using multi-omics and system biology approaches is needed for the discovery of genes governing important agronomic traits. Forward/reverse genetics are required for their functional validation so that those important genes can be utilized for genetic improvement of rice.

The ultimate goal of rice functional genomics studies is to identify genes controlling important agronomic traits and to explore them for the genetic improvement of rice. The project ‘Rice 2020’ was proposed by International Rice Functional Genomics Consortium for a coordinated effort at the international level to decipher the biological function of each gene present in the rice genome by the year 2020 [38]. World population is projected to reach 9 billion by 2042. At the tenth ‘International Symposium of Rice Functional Genomics’ held in Thailand in 2012, the emphasis was again given on coordinated efforts towards new discoveries in rice biology for rice improvement to address the problem of food security for the 9 billion people. Rice (*Oryza sativa* spp. *japonica* cv. Nipponbare) genome was sequenced in 2005 (IRGSP 2005). Currently, genomic sequences of more than 4000 genotypes including wild and cultivated rice varieties are available in public domain. The big challenge of the post-genomic era is to progressively investigate the role of all the genes (Figure 3). Currently, more than 3200 genes belonging to different families and performing diverse functions have been cloned and characterized [46]. A straight and effective method to discover the function of a gene is to silence or activate it by mutagenesis and to compare the phenotype of created mutant with wild type plant by means of forward and reverse genetics approaches.

Insertion mutagenesis in rice started in the late 1990s at the Pohang University of Technology (POSTECH), South Korea [47] and at the National Institute of Agrobiological Sciences (NIAS), Japan [48], using T-DNA and endogenous Tos17 retrotransposons, respectively. A large number of mutant lines have been generated by these two institutes. In fact, among all the insertional mutant resources, POSTECH, South Korea has the largest collection of indexed mutants with 99,559 flanking sequence tags (FSTs) mapped to RGAP v7 (Rice Genome Annotation Project version 7) annotation [37,38,47] (Table 1). Among worldwide mutant collections, six of them have been generated employing T-DNA (Table 1). After POSTECH, Rice Mutant Database (RMD), China stands second and contains 91,792 mapped FSTs lines generated by T-DNA enhancer trap method [18]. These mutant lines have been comprehensively shared and distributed among different laboratories in the world to decode the function of diverse genes. Few other important T-DNA mutant libraries were created by Hua Zhong Agricultural University, China [3], Beijing Biotechnology Research Institute, Chinese Academy of Agricultural Sciences (CAAS), China [44], the Shanghai Institute of Plant Physiology and Ecology, China and Génoplante consortium, France, involving private (Biogemma and Bayer Crop Science) and public research laboratories (The French Agricultural Research Centre for International Development, National Institute for Agricultural Research, National Centre for Scientific Research and Institute for Development Research) [41,42], and by Institute of Plant and Microbial Biology, Academia Sinica, Taiwan [40]. Simultaneously, maize transposons like Spm and Ds have been utilized for mutagenesis in many countries including Korea (Plant Molecular Biology and Biotechnology Research Center [21]), Europe [43,49], Singapore, and the United States [6,50]. Several strategies, such as activation tagging, enhancer trap, gene traps, and knockout have been used to generate these mutant resources. Information from different rice mutant resources have been integrated and have been put into two databases, OryGenesDB (http://orygenesdb.cirad.fr) and RiceGE (http://signal.salk.edu/cgi-bin/RiceGE) [51]. Moreover, the RiceGE database was updated in 2018 on the basis of MSU (Michigan State University) rice genome annotation project release 7. Most of the FSTs can be searched at these two databases. As per the latest update (2018), RiceGE contains 382,266 entries of mutants. Most of the available mutant libraries available are originated from *japonica* rice varieties such as Dongjin, Hwayoung, Kitaake, Nipponbare, Tainung 67, Zhonghua 11, Zhonghua 15, etc. There are some collections of mutants also available in *indica* rice varieties, such as IR-64, Kasalath, and SSBM. Many *indica* mutants have also been generated using physical (Fast neutron, γ-ray) and chemical (EMS) mutagens. It is worth mentioning here that *indica* rice accounts for 80% of the global rice production in terms of cultivation area or yield. However, most of the insertional mutant resources are available in *japonica* rice varieties; this may be due to the fact that *japonica* varieties are easily transformed by agrobacterium mediated protocol whereas *indica* varieties are difficult genotypes with poor transformation efficiency.

Regardless of the type of insertional mutagens, the insertions are unevenly dispersed along all the rice chromosomes. Insertion frequencies are higher in euchromatin regions (hot spots), especially in the telomeric and sub-telomeric regions of the chromosomes in comparison to heterochromatic regions (cold spots) near the centromere regions. Regarding the genome-wide coverage of T-DNA insertions, they have low occurrence in repetitive DNA regions. 48–63% of T-DNA insertions are found in intergenic regions. Furthermore, they show clear bias toward the 5’ upstream and 3’ downstream regions of genes [38,45]. Most importantly, about half of the nuclear genome and 60% of total nuclear genes of rice have been mutated [33].

T-DNA mutants are genetically modified (GM) in nature as they are generated via the agrobacterium-mediated transformation method. This causes two limitations; firstly, somaclonal variations are induced due to the tissue culture work, and secondly, field multiplication and the distribution process of mutant seeds become complicated under tight rules and regulations in different countries. When such germplasms are exchanged, material transfer agreement, phytosanitary certificate, and import permits are required. Seeds are also tested by standard quarantine procedures to eradicate seed-borne pathogens. These things make the procedure complicated and cause a delay in seed delivery. Furthermore, different scientific groups in the world employ different systems to describe traits and phenotypic variations of mutants, which make cataloging of phenotypes very non-uniform. A uniform system/methodology should be adopted worldwide to compare mutants. Good facilities are also required for phenomics analysis of mutants along with proper storage and multiplication of seeds. Nottingham Arabidopsis Stock Centre (NASC) is an international centre for the proper storage, maintenance, and distribution of Arabidopsis seeds. Similarly, a centralized stock centre for rice mutant collections should be established.

As with other mutagenesis methods, induced insertional mutagenesis approach can also generate novel phenotypes. However, in these insertional mutants, the relationship between genotype to phenotype is very low (5–10%) [52,53]. This phenomenon is due to spontaneous outcrossing occurring in different generations in the rice fields [54]. While screening for novel phenotypes in Taiwan Rice Insertional Mutagenesis (TRIM) population, Chang et al. 2019, identified a semi-sterile mutant (*sstl*), however, it was later found to be associated with an un-tagged T-DNA line [55].

Apart from creating loss of function mutants, another strategy for identification of the function of important genes is to generate gain of function mutations. It is accomplished by Functional Overexpression (FOX) of full-length cDNAs library. This method is popularly known as the FOX gene hunting system. Around 12,000 rice FOX lines were generated by the National Institute of Agrobiological Sciences, Japan [56]. In the same institute, Kondou’s lab used Arabidopsis for heterologous expression of full-length rice cDNAs. They generated a library of around 23,000 independent transgenic plants. Systematic screening of these lines under various conditions led to the identification of the function of diverse rice genes. Later, [57] also generated more than 30,000 Arabidopsis FOX lines overexpressing rice full-length cDNA. They also developed a database ‘RiceFOX’ in which a broad range of information regarding traits and corresponding genes was incorporated [57].

Recently, a great tool for genome editing named ‘clustered regularly interspaced short palindromic repeats-associated nuclease 9 (CRISPR/Cas9) system’ has been developed. It has been used for targeted mutagenesis in several crops and other organisms [20]. Using this tool, genome-wide mutant libraries with highly efficient targeted editing of rice genes have been created [58,59]. In comparison to the traditional methods of mutagenesis, this system is speedy, economical, and causes pinpoint mutations using the guide RNA sequence of the target gene. Additionally, screening for phenotypes linked to lethal genes is possible in the T0 generation using this tool, which is a drawback of insertional heterozygous mutants. In the future, improvement in this technology will further build up the mutant resources accessible for functional genomics research.

## 8. Conclusions

Development of improved and climate-change ready super-rice relies on the identification and functional validation of important genes related to agronomic traits like stress tolerance and yield (Figure 3). Mutant libraries generated by insertional mutagenesis have facilitated key gene discoveries. Recently evolved CRISPR technology has also speed-up the generation and screening of rice mutants for valuable traits. However, after creating genome-wide mutant pools, confirmation of the cause and effect correlation between the corresponding mutation in the genotype and the resulting phenotype continues to be challenging in forward/reverse genetics studies. Another obstacle is gene duplication, which causes functional redundancy and results in a nonappearance of noticeable phenotypic alteration. For these reasons, systematic screening systems well-equipped with phenomics facilities are required to be established. A user-friendly and integrative network/platform housing all the mutant resources, system biology tools, and easily interpretable and accessible omics data, is also needed so that all the insights sought by scientists can be provided to boost functional genomics discoveries (Figure 3).

## Figures and Tables

**Table 1:** T-DNA mutant resources in rice.

Resource/Database Name	Institution	Genotype	Type of T-DNA Mutagen	Number of Available FSTs Lines ^a^	Number of Mapped FSTs ^a^	Website of the Resource/Database	Reference	Number of Citations ^b^
POSTECH Rice Insertion Database (RISD)	Pohang University of Science and Technology, South Korea	Dongjin Hwayoung Kitaake	Gene trap, Activation tagging	107,171	99,559	http://cbi.khu.ac.kr/	[36] [37] [38]	684 94 452 308
Rice Mutant Database (RMD)	Huazhong Agricultural University, China	Zhonghua 11 Zhonghua 15 Nipponbare	Enhancer trap, Tos17	85,315	91,792	http://rmd.ncpgr.cn/	[18] [39]	251 183
Taiwan Rice Insertion Mutant (TRIM)	Institute of Plant and Microbial Biology, Academia Sinica, Taiwan	Tainung 67	Activation tagging	59,804	58,764	http://trim.sinica.edu.tw	[40]	192
Oryza Tag Line (OTL) Génoplante	CIRAD-INRA-IRD-CNRS, France	Nipponbare	Enhancer trap, Tos17	29,263	29,429	http://oryzatagline.cirad.fr/	[41] [42]	268 104
Shanghai T-DNA Insertion Population Database (SHIP)	Shanghai Institute of Plant Physiology and Ecology (SIPPE), China	Zhonghua 11	Enhancer trap	10,381	10,934	http://www.plantsignal.cn/	[43]	42
Chinese Academy of Agricultural Sciences (CAAS)	Beijing Biotechnology Research Institute, CAAS, China	Nipponbare	Activation tagging	N/A	N/A	N/A	[44]	85

^a^ numbers are quoted from the SIGnAL website of the SALK Institute (http://signal.salk.edu/Source/AtTOME_Data_Source.html). ^b^ numbers are quoted from the google scholar website (https://scholar.google.co.in/).N/A- Not available.

## References

[1] Sikora P., Chawade A., Larsson M., Olsson J., Olsson O. (2011). Mutagenesis as a tool in plant genetics, functional genomics, and breeding. Int. J. Plant Genom..

[2] Kodym A., Afza R. (2003). Physical and chemical mutagenesis. Methods Mol. Biol..

[3] Wu C., Li X., Yuan W., Chen G., Kilian A., Li J., Xu C., Li X., Zhou D.X., Wang S. (2003). Development of enhancer trap lines for functional analysis of the rice genome. Plant J..

[4] Mba C., Afza R., Bado S., Jain S.M. (2010). Induced Mutagenesis in Plants Using Physical and Chemical Agents. Plant Cell Culture: Essential Methods.

[5] Krishnan A., Guiderdoni E., An G., Hsing Y.C., Han C.D., Lee M.C., Yu S.M., Upadhyaya N., Ramachandran S., Zhang Q. (2009). Mutant Resources in Rice for Functional Genomics of the Grasses. PLANT Physiol..

[6] Maarse H. (2017). Rice. Volatile Compounds in Foods and Beverages.

[7] Lawrence C.W. (1991). Classical mutagenesis techniques. Methods Enzymol..

[8] Krieg D.R. (1963). Ethyl Methanesulfonate-Induced Reversion Of Bacteriophage T4rii Mutants. Genetics.

[9] Till B.J., Reynolds S.H., Weil C., Springer N., Burtner C., Young K., Bowers E., Codomo C.A., Enns L.C., Odden A.R. (2004). Discovery of induced point mutations in maize genes by TILLING. BMC Plant Biol..

[10] Comai L., Henikoff S. (2006). TILLING: Practical single-nucleotide mutation discovery. Plant J..

[11] Kumawat A., Gupta N.K., Raj Jain N., Nayama S. (2019). Studies on the Effect of Plant Growth Regulators and Micronutrients on Okra (Abelmoschus esculentus L) cv. Parbhani Kranti. Int. J. Curr. Microbiol. Appl. Sci..

[12] Kim S.Y., Kim C.K., Kang M., Ji S.U., Yoon U.H., Kim Y.H., Lee G.S. (2018). A Gene Functional Study of Rice Using Ac/Ds Insertional Mutant Population. Plant Breed. Biotech..

[13] Moin M., Bakshi A., Madhav M.S., Kirti P.B. (2018). Cas9/sgRNA-based genome editing and other reverse genetic approaches for functional genomic studies in rice. Brief. Funct. Genom..

[14] Springer P.S. (2000). Gene traps: Tools for plant development and genomics. Plant Cell.

[15] Wu J.-L., Wu C., Lei C., Baraoidan M., Bordeos A., Madamba M.R.S., Ramos-Pamplona M., Mauleon R., Portugal A., Ulat V.J. (2005). Chemical- and irradiation-induced mutants of indica rice IR64 for forward and reverse genetics. Plant Mol. Biol..

[16] Hwang H.-H., Yu M., Lai E.M. (2017). Agrobacterium-Mediated Plant Transformation: Biology and Applications. Arab. Book.

[17] Sun Q., Zhou D.X. (2008). Rice jmjC domain-containing gene JMJ706 encodes H3K9 demethylase required for floral organ development. Proc. Natl. Acad. Sci. USA.

[18] Wu H., Sparks C., Amoah B., Jones H.D. (2003). Factors influencing successful Agrobacterium-mediated genetic transformation of wheat. Plant Cell Rep..

[19] Dubin M.J., Mittelsten Scheid O., Becker C. (2018). Transposons: A blessing curse. Curr. Opin. Plant Biol..

[20] Babakhani S., Oloomi M. (2018). Transposons: The agents of antibiotic resistance in bacteria. J. Basic Microbiol..

[21] An G., Lee S., Kim S.H., Kim S.R. (2005). Molecular genetics using T-DNA in rice. Plant Cell Physiol..

[22] Munoz-Lopez M., Garcia-Perez J. (2010). DNA Transposons: Nature and Applications in Genomics. Curr. Genom..

[23] Kolesnik T., Szeverenyi I., Bachmann D., Kumar C.S., Jiang S., Ramamoorthy R., Cai M., Ma Z.G., Sundaresan V., Ramachandran S. (2004). Establishing an efficient Ac/Ds tagging system in rice: Large-scale analysis of Ds flanking sequences. Plant J..

[24] Ramamoorthy R., Jiang S.Y., Ramachandran S. (2011). Oryza sativa cytochrome P450 family member OsCYP96B4 reduces plant height in a transcript dosage dependent manner. PLoS ONE.

[25] Jiang N., Bao Z., Zhang X., Hirochika H., Eddy S.R., McCouch S.R., Wessler S.R. (2003). An active DNA transposon family in rice. Nature.

[26] Kikuchi K., Terauchit K., Wada M., Hirano H.Y. (2003). The plant MITE mPing is mobilized in anther culture. Nature.

[27] Nakazaki T., Okumoto Y., Horibata A., Yamahira S., Teraishi M., Nishida H., Inoue H., Tanisaka T. (2003). Mobilization of a transposon in the rice genome. Nature.

[28] Hancock C.N., Zhang F., Floyd K., Richardson A.O., LaFayette P., Tucker D., Wessler S.R., Parrott W.A. (2011). The Rice Miniature Inverted Repeat Transposable Element mPing Is an Effective Insertional Mutagen in Soybean. Plant Physiol..

[29] Teramoto S., Tsukiyama T., Okumoto Y., Tanisaka T. (2014). Early Embryogenesis-Specific Expression of the Rice Transposon Ping Enhances Amplification of the MITE mPing. PLoS Genet..

[30] Ram H., Kaur A., Gandass N., Singh S., Deshmukh R., Sonah H., Sharma T.R. (2019). Molecular characterization and expression dynamics of MTP genes under various spatiotemporal stages and metal stress conditions in rice. PLoS ONE.

[31] Hirochika H. (1997). Retrotransposons of rice: Their regulation and use for genome analysis. Plant Mol. Biol..

[32] Chin H.G., Choe M.S., Lee S.H., Park S.H., Park S.H., Koo J.C., Kim N.Y., Lee J.J., Oh B.G., Yi G.H. (1999). Molecular analysis of rice plants harboring an Ac/Ds transposable element-mediated gene trapping system. Plant J..

[33] Jung K.-H., Hur J., Ryu C.-H., Choi Y., Chung Y.-Y., Miyao A., Hirochika H., An G. (2003). Characterization of a rice chlorophyll-deficient mutant using the T-DNA gene-trap system. Plant Cell Physiol..

[34] Hanin M., Paszkowski J. (2003). Plant genome modification by homologous recombination. Curr. Opin. Plant Biol..

[35] Feldmann K.A. (1991). T—DNA insertion mutagenesis in Arabidopsis: Mutational spectrum. Plant J..

[36] Jeon J.S., Lee S., Jung K.H., Jun S.H., Jeong D.H., Lee J., Kim C., Jang S., Lee S., Yang K. (2000). T-DNA insertional mutagenesis for functional genomics in rice. Plant J..

[37] Ryu C.H., You J.H., Kang H.G., Hur J., Kim Y.H., Han M.J., An K., Chung B.C., Lee C.H., An G. (2004). Generation of T-DNA tagging lines with a bidirectional gene trap vector and the establishment of an insertion-site database. Plant Mol. Biol..

[38] Jeong D.H., An S., Park S., Kang H.G., Park G.G., Kim S.R., Sim J., Kim Y.O., Kim M.K., Kim S.R. (2006). Generation of a flanking sequence-tag database for activation-tagging lines in japonica rice. Plant J..

[39] Zhang J. (2005). RMD: A rice mutant database for functional analysis of the rice genome. Nucleic Acids Res..

[40] Hsing Y.I., Chern C.G., Fan M.J., Lu P.C., Chen K.T., Lo S.F., Sun P.K., Ho S.L., Lee K.W., Wang Y.C. (2007). A rice gene activation/knockout mutant resource for high throughput functional genomics. Plant Mol. Biol..

[41] Sallaud C., Gay C., Larmande P., Bès M., Piffanelli P., Piégu B., Droc G., Regad F., Bourgeois E., Meynard D. (2004). High throughput T-DNA insertion mutagenesis in rice: A first step towards in silico reverse genetics. Plant J..

[42] Johnson A.A.T., Hibberd J.M., Gay C., Essah P.A., Haseloff J., Tester M., Guiderdoni E. (2005). Spatial control of transgene expression in rice (*Oryza sativa* L.) using the GAL4 enhancer trapping system. Plant J..

[43] Van Enckevort L.J.G., Droc G., Piffanelli P., Greco R., Gagneur C., Weber C., González V.M., Cabot P., Fornara F., Berri S. (2005). EU-OSTID: A collection of transposon insertional mutants for functional genomics in rice. Plant Mol. Biol..

[44] Wan S., Wu J., Zhang Z., Sun X., Lv Y., Gao C., Ning Y., Ma J., Guo Y., Zhang Q. (2009). Activation tagging, an efficient tool for functional analysis of the rice genome. Plant Mol. Biol..

[45] Krysan P.J., Young J.C., Sussman M.R. (1999). T-DNA as an insertional mutagen in Arabidopsis. Plant Cell.

[46] Yao W., Li G., Yu Y., Ouyang Y. (2018). FunRiceGenes dataset for comprehensive understanding and application of rice functional genes. Gigascience.

[47] Jeong D.-H. (2002). T-DNA Insertional Mutagenesis for Activation Tagging in Rice. Plant Physiol..

[48] Miyao A. (2003). Target Site Specificity of the Tos17 Retrotransposon Shows a Preference for Insertion within Genes and against Insertion in Retrotransposon-Rich Regions of the Genome. Plant Cell.

[49] Upadhyaya N.M., Zhu Q.H., Zhou X.R., Eamens A.L., Hoque M.S., Ramm K., Shivakkumar R., Smith K.F., Pan S.T., Li S. (2006). Dissociation (Ds) constructs, mapped Ds launch pads and a transiently-expressed transposase system suitable for localized insertional mutagenesis in rice. Theor. Appl. Genet..

[50] Kumar C.S., Wing R.A., Sundaresan V. (2005). Efficient insertional mutagenesis in rice using the maize En/Spm elements. Plant J..

[51] Droc G., Périn C., Fromentin S., Larmande P. (2009). OryGenesDB 2008 update: Database interoperability for functional genomics of rice. Nucleic Acids Res..

[52] Droc G., An G., Wu C., Hsing Y.C., Hirochika H., Pereira A., Sundaresan V., Upadhyaya N., Ramachandran S., Comai L., Zhang Q., Wing R.A. (2013). Mutant Resources for Functional Analysis of the Rice Genome. Genetics and Genomics of Rice. Plant Genetics and Genomics: Crops and Models.

[53] Wei F.J., Droc G., Guiderdoni E., Hsing Y.I. (2013). International Consortium of Rice Mutagenesis: Resources and beyond. Rice (NY).

[54] Wei F., Tsai Y., Hsu Y., Chen Y., Huang C., Wu H., Huang L., Lai M., Kuang L., Yu S. (2016). Lack of genotype and phenotype correlation in a rice T-DNA tagged line is likely caused by introgression in the seed source. PLoS ONE.

[55] Chang C.L., Serapion J.C., Hung H.H., Lin Y.C., Tsai Y.C., Jane W.N., Chang M.C., Lai M.H., Hsing Y.C. (2019). Studies of a rice sterile mutant sstl from the TRIM collection. Bot Stud..

[56] Nakamura H., Hakata M., Amano K., Miyao A., Toki N., Kajikawa M., Pang J., Higashi N., Ando S., Toki S. (2007). A genome-wide gain-of-function analysis of rice genes using the FOX-hunting system. Plant Mol. Biol..

[57] Sakurai T., Kondou Y., Akiyama K., Kurotani A., Higuchi M., Ichikawa T., Kuroda H., Kusano M., Mori M., Saitou T. (2011). RiceFOX: A database of Arabidopsis mutant lines overexpressing rice full-length cDNA that contains a wide range of trait information to facilitate analysis of gene function. Plant Cell Physiol..

[58] Meng X., Yu H., Zhang Y., Zhuang F., Song X., Gao S., Gao C., Li J. (2017). Construction of a Genome-Wide Mutant Library in Rice Using CRISPR/Cas9. Mol. Plant.

[59] Lu Y., Zhu J.K. (2017). Precise Editing of a Target Base in the Rice Genome Using a Modified CRISPR/Cas9 System. Mol. Plant.

